# Gram-Negative Taxa and Antimicrobial Susceptibility after Fecal Microbiota Transplantation for Recurrent Clostridioides difficile Infection

**DOI:** 10.1128/mSphere.00853-20

**Published:** 2020-10-14

**Authors:** Danielle Barrios Steed, Tiffany Wang, Divyanshu Raheja, Alex D. Waldman, Ahmed Babiker, Tanvi Dhere, Colleen S. Kraft, Michael H. Woodworth

**Affiliations:** a Emory University School of Medicine, Atlanta, Georgia, USA; b Emory University School of Medicine, Department of Pathology and Laboratory Medicine, Atlanta, Georgia, USA; c Emory University School of Medicine, Department of Medicine, Division of Digestive Diseases, Atlanta, Georgia, USA; d Emory University School of Medicine, Department of Medicine, Division of Infectious Diseases, Atlanta, Georgia, USA; University of Michigan-Ann Arbor

**Keywords:** FMT, fecal microbiota transplant, recurrent infection, *C. difficile* infection, infection, microbiome, Gram negative

## Abstract

Fecal microbiota transplantation (FMT) has promising applications in reducing multidrug-resistant organism (MDRO) colonization and antibiotic resistance (AR) gene abundance. However, data on clinical microbiology results after FMT are limited. We examined the changes in antimicrobial susceptibility profiles in patients with Gram-negative infections in the year before and the year after treatment with FMT for recurrent Clostridioides difficile infection (RCDI). We also examined whether a history of FMT changed health care provider behavior with respect to culture ordering and antibiotic prescription. Medical records for RCDI patients who underwent FMT at Emory University between July 2012 and March 2017 were reviewed retrospectively. FMT-treated patients with Gram-negative culture data in the 1-year period preceding and the 1-year period following FMT were included. Demographic and clinical data were abstracted, including CDI history, frequency of Gram-negative cultures, microbiological results, and antibiotic prescription in response to positive cultures in the period following FMT. Twelve patients were included in this case series. We pooled data from infections at all body sites and found a decrease in the number of total and Gram-negative cultures post-FMT. We compared susceptibility profiles across taxa given the potential for horizontal transmission of AR elements and observed increased susceptibility to nitrofurantoin, trimethoprim-sulfamethoxazole, and the aminoglycosides. FMT did not drastically influence health care provider ordering of bacterial cultures or antibiotic prescribing practices. We observed a reduction in Gram-negative cultures and a trend toward increased antimicrobial susceptibility. This study supports further investigation of FMT as a means of improving antimicrobial susceptibility.

**IMPORTANCE** Fecal microbiota transplantation (FMT), which is highly efficacious in treating recurrent C. difficile infection (RCDI), has a promising application in decolonization of multidrug-resistant organisms, reduction of antibiotic resistance gene abundance, and restoration of healthy intestinal microbiota. However, data representing clinical microbiology results after FMT are limited. We sought to characterize the differences in culture positivity and antimicrobial susceptibility profiles in patients with Gram-negative infections in the year before and the year after FMT for RCDI. Drawing on prior studies that had demonstrated the success of FMT in eradicating extraintestinal infections and the occurrence of patient-level interspecies transfer of resistance elements, we employed an agnostic analytic approach of reviewing the data irrespective of body site or species. In a small RCDI population, we observed an improvement in the antimicrobial susceptibility profile of Gram-negative bacteria following FMT, which supports further study of FMT as a strategy to combat antibiotic resistance.

## Abstract (precis)

Fecal microbiota transplantation (FMT), which is highly efficacious in treating recurrent C. difficile infection (RCDI), has a promising application in decolonization of multidrug-resistant organisms, reduction of antibiotic resistance gene abundance, and restoration of healthy intestinal microbiota. However, data representing clinical microbiology results after FMT are limited. We sought to characterize the differences in culture positivity and antimicrobial susceptibility profiles in patients with Gram-negative infections in the year before and the year after FMT for RCDI. Drawing on prior studies that had demonstrated the success of FMT in eradicating extraintestinal infections and the occurrence of patient-level interspecies transfer of resistance elements, we employed an agnostic analytic approach of reviewing the data irrespective of body site or species. In a small RCDI population, we observed an improvement in the antimicrobial susceptibility profile of Gram-negative bacteria following FMT, which supports further study of FMT as a strategy to combat antibiotic resistance.

## INTRODUCTION

Antimicrobial resistance (AR) is a major global threat (1–4) with few viable solutions to meet this growing need. Infection with multidrug-resistant organisms (MDROs) is associated with increased morbidity and mortality (5, 6), and the U.S. Centers for Disease Control and Prevention estimates that over 35,000 people die from AR bacterial infections annually (7). In some cases, MDRO isolates were been identified that were resistant to all available antibiotics. Novel therapeutic strategies to address AR are urgently needed. One potential strategy with increasingly recognized potential is targeting the gut microbiome as a determinant of MDRO colonization and infection risk.

Abnormal microbiome states such as a high relative abundance of pathogens, low diversity in typically diverse microbial communities, and unfavorable shifts in microbial metabolic function are often described as dysbiosis (8, 9). Decreased intestinal microbiome diversity often correlates with loss of taxa that provide colonization resistance against MDROs such as Clostridioides difficile and multidrug resistant Gram-negative bacteria. This perturbation to the gut microbiome can be prolonged (10), and MDROs have been shown to have protracted colonization approaching a median of 1 year in multiple studies (11–13).

Fecal microbiota transplantation (FMT) has emerged as a promising therapy for MDRO decolonization. It is highly efficacious in treating recurrent C. difficile infection (RCDI), with resolution rates of approximately 90% (14, 15), and multiple groups have observed decreased MDRO colonization and infection after FMT. FMT has been shown to immediately increase intestinal microbial diversity and increase colonization resistance against pathogenic bacteria (16–18).

The human gut microbiome also constitutes a major reservoir of AR genes (19–21). Bacterial populations attain resistance either through genetic mutation or through horizontal transfer. The lower gastrointestinal (GI) tract microbiome is characterized by high cell density and potential for biofilm formation. These features facilitate cell-to-cell contact, which enables horizontal gene transmission and dissemination of AR determinants (22, 23). Case reports and case series have demonstrated resistance via inter- and intraspecies plasmid transfer at the patient level. An analysis of a nosocomial outbreak of carbapenem-resistant *Enterobacteriaceae* (CRE) found evidence supporting plasmid transfer among Klebsiella pneumoniae, Enterobacter cloacae, and Citrobacter freundii, presumably in hospital sinks, using single-molecule sequencing (24). Another case report highlighted the rare occurrence of a *bla*_VIM-1_ (carbapenem resistance)-expressing Aeromonas hydrophila strain in a patient who had suffered prolonged submersion; after the patient was later found to harbor three *Enterobacteriaceae* species, all of which expressed the same plasmid, the mutation was presumed to have been conferred via horizontal gene transfer (25). Within the GI tract itself, probable conjugal transfer of an OXA-48 (carbapenem resistance)-encoding plasmid from E. cloacae to other members of *Enterobacteriaceae* (26) and transfer of ampicillin resistance between different Escherichia coli strains (27) as well as presumed transfer of a MDR plasmid from K. pneumoniae to E. coli have been reported previously (28). Regardless of the origins of these resistance determinants, FMT has been shown to reduce the diversity and number of AR genes in recipient microbiota (29).

Despite FMT’s promising application in the decolonization of multidrug-resistant organisms, reduction of antibiotic resistance genes, and restoration of healthy intestinal microbiota, the data on clinical culture and susceptibility results after FMT are limited. As bacterial culture is the clinical gold standard for identification and susceptibility testing, we sought to estimate differences in culture positivity and clinical antimicrobial susceptibility profiles in a series of patients with Gram-negative infections in the year before and the year after treatment with FMT for RCDI. We applied a novel approach of agnostic Gram-negative antimicrobial susceptibility analysis to detect changes in the antimicrobial susceptibility profile at the microbial community level given the potential for horizontal transmission of AR genetic determinants among gut taxa (22, 23) and the expected resistome changes after FMT. As part of our analysis to determine factors contributing to the reduction of cultures post-FMT, we also examined whether a history of FMT influenced health care provider culture ordering and antibiotic prescription behavior in the post-FMT study period.

## RESULTS

### Patients and culture results.

**FIG 1:**
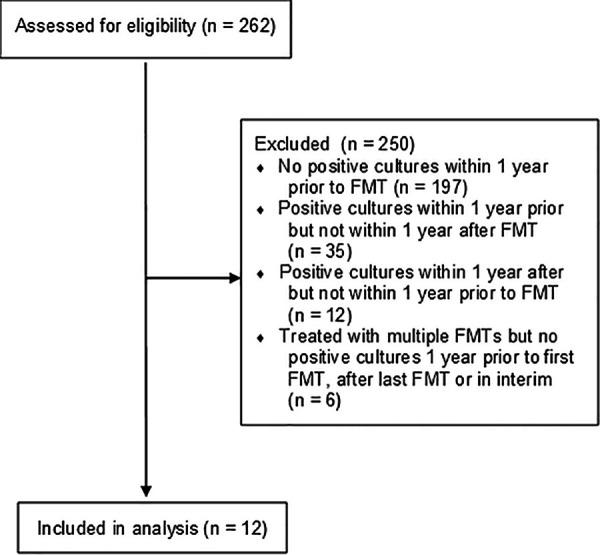
Flow diagram illustrating patients assessed for eligibility, reasons for exclusion from analysis, and number included for analysis.

Among the 262 patients reviewed during the study period who underwent FMT for recurrent CDI, 12 were identified with Gram-negative culture data in the periods of 1 year preceding and 1 year following FMT and were included in the case series (Fig. 1). Demographic and clinical data are summarized in Table 1. The majority of the patients had had at least 3 previous episodes of CDI. The majority of the patients were female (66.7%), and the mean age at the time of treatment was 64.8 years (range, 45 to 80 years). Two patients (subject identifiers [IDs] 2 and 3) underwent two FMTs but had no intervening Gram-negative culture data available from the period between their two procedures, and culture data were collected during 1 year preceding their first FMT and 1 year following their last FMT.

**TABLE 1:** Clinical characteristics of recurrent Clostridioides difficile patients with Gram-negative microbiological data 1 year before and 1 year after FMT from 2012 to 2017^*a*^

Subject ID	Age (yrs)	Sex	Race	Prior medical history/comorbid conditions	Prior procedural/surgical history	No. of incidences of CDI	Treatment(s) for CDI prior to study period FMTs	Route of FMT/FMT no.	Donor	Outcome	Adverse event	Institutional follow-up
1	80	F	Caucasian	Recurrent UTIs, HTN, GERD, CVA, DLD, periodic limb movements, OA	Laparoscopic cholecystectomy, hysterectomy	Unknown total no.—denoted as multiple	Multiple oral vancomycin tapers	Nasogastric tube/1	Friend	Remission		Until 17 mo post-FMT, patient subsequently moved to Florida
												
2	73	F	African American	Churg-Strauss syndrome, pulmonary fibrosis, Zenker’s diverticulum, right breast cancer, papillary thyroid cancer, HTN, chronic systolic HF, hypothyroidism, lumbar stenosis, osteoporosis, DM type 2, DLD	Thyroidectomy, right lumpectomy, hysterectomy	Unknown total no.—denoted as multiple	Multiple vancomycin tapers, fidaxomicin, two fecal transplants	Nasogastric tube/1; nasogastric tube/2	Daughter	Improvement in symptoms for 6 wks, then CDI relapse		Until 4 mo post-FMT, subsequently elected inpatient hospice
												
3	71	F	African American	DDRT for hypertensive nephrosclerosis, recurrent UTIs, right lower extremity DVT, vertebral osteomyelitis, CVA, depression	DDRT, IVC filter placement, cholecystectomy, hysterectomy, carotid endarterectomy	8	Multiple courses of oral vancomycin, prolonged vancomycin taper, vancomycin + rifaximin	Nasogastric tube/1; colonoscopy/2	Daughter	Remission after 2nd fecal transplant		Until 28 mo post-FMT, after which point patient noted to be deceased
												
68	66	F	Caucasian	Metastatic myxoid sarcoma of the pelvis complicated by enterovesical fistula	Tumor debulking, multiple small-bowel resections, hysterectomy, appendectomy, bladder resection, and small-bowel resection	3	Oral metronidazole, oral vancomycin	Colonoscopy/1	Fecal DON 2014-01	Continued but less frequent diarrhea, subsequent C. difficile testing negative		Until 20 mo post-FMT, subsequently elected hospice care
												
79	50	M	Caucasian	DDRT recipient, DM type 2 complicated by retinopathy, nephropathy, neuropathy, bladder neck stricture, recurrent UTIs, CAD, PVD, asthma, OSA, HTN, prostate cancer, testicular cancer	DDRT, 4-vessel CABG, PTCA and stent placement, radical prostatectomy, orchiectomy, right tibia/fibula ORIF, left upper extremity AV graft creation, cholecystectomy	>5	Oral vancomycin, oral metronidazole	Colonoscopy/1	Fecal DON 2013-10	Remission		Until present-day
												
92	75	F	Caucasian	Parkinson’s disease, recurrent UTIs, anemia, hypothyroidism, OA, erosive esophagitis	Deep brain stimulator implantation, bilateral knee replacement surgeries	4	i.v. and oral metronidazole, oral vancomycin, oral vancomycin + metronidazole, fidaxomicin + oral vancomycin taper	Colonoscopy/1	Fecal DON 2013-10	Remission	Increased flatulence	Until 48 mo post-FMT
												
166	45	F	Caucasian	DDRT recipient × 3 due to FSGS, recurrent UTIs, CVA, osteoporosis, OA	DDRT, ex-lap and small-bowel resection, cholecystectomy	>3	Oral metronidazole, oral vancomycin, oral vancomycin taper, fecal transplant	Colonoscopy/1	Fecal DON 2014-2	No improvement in symptoms		Until present-day
169	74	M	Caucasian	COPD, PE, atrial fibrillation, CAD, esophageal perforation	Cervical disc fusion, esophageal perforation repair, IVC filter placement and removal, PEG placement	3	IV metronidazole, oral vancomycin, oral + rectal vancomycin	Colonoscopy/1	Fecal DON 2014-2	Remission	Transient constipation	Until 7 mo post-FMT
												
202	50	F	Caucasian	Seizures, irritable bowel syndrome, self-reported Crohn’s disease and celiac sprue	Kyphoplasty	Unknown total no.—self-reported as >10–12	Oral metronidazole, oral vancomycin	Colonoscopy/1	Fecal DON 2014-1	Continued diarrhea		Until 23 mo post-FMT
												
226	48	F	Caucasian	Crohn’s disease, DM type 2, dyslipidemia, depression, anxiety, GERD, PCOS, gastroparesis	Sigmoid resection, proctectomy, end colostomy, rectovaginal fistula repair	3	Unknown	Colonoscopy/1	Fecal DON 2014-1	Continued diarrhea		Until present-day
												
230	65	M	Caucasian	T3N2 rectal cancer, atrial fibrillation, prostate cancer, CAD, genital herpes simplex, urinary retention complicated by recurrent UTIs	Brachytherapy, external beam radiation therapy, proctectomy with coloanal anastomosis and diverting loop ileostomy with subsequent loop ileostomy reversal, PCI with stent placement	3	Oral metronidazole, oral vancomycin, oral vancomycin taper	Colonoscopy/1	Fecal DON 2014-1	Remission	Transient constipation	Until present-day
												
232	80	M	African American	Multiple myeloma, myelodysplastic syndrome, colon cancer, CAD, chronic systolic HF, necrotizing cellulitis of the left leg, CKD stage IV	Hemicolectomy	4	Oral vancomycin + i.v. metronidazole, oral vancomycin	Colonoscopy/1	Fecal DON 2014-1	Remission	Transient diarrhea	Until 20 mo post-FMT, subsequently elected hospice care and noted to be deceased shortly thereafter

a Abbreviations: AV, arteriovenous; CABG, coronary artery bypass graft; CAD, coronary artery disease; CKD, chronic kidney disease; COPD, chronic obstructive pulmonary disorder; CVA, cerebrovascular accident; DON, donor (the numbers after “DON” are the year of sample collection and the numerical identifier specific to each donor); DDRT, deceased donor renal transplant; DLD, dyslipidemia; DM, diabetes mellitus; DON, donor; DVT, deep vein thrombosis; ex-lap, exploratory laparotomy; F, female; FSGS, focal segmental glomerulosclerosis; GERD, gastroesophageal reflux disease; HF, heart failure; HTN, hypertension; i.v., intravenous; IVC, inferior vena cava; M, male; OA, osteoarthritis; ORIF, open reduction internal fixation; PE, pulmonary embolus; PEG, percutaneous endoscopic gastrostomy; PTCA, percutaneous transluminal coronary angioplasty; UTI, urinary tract infection.

**FIG 2:**
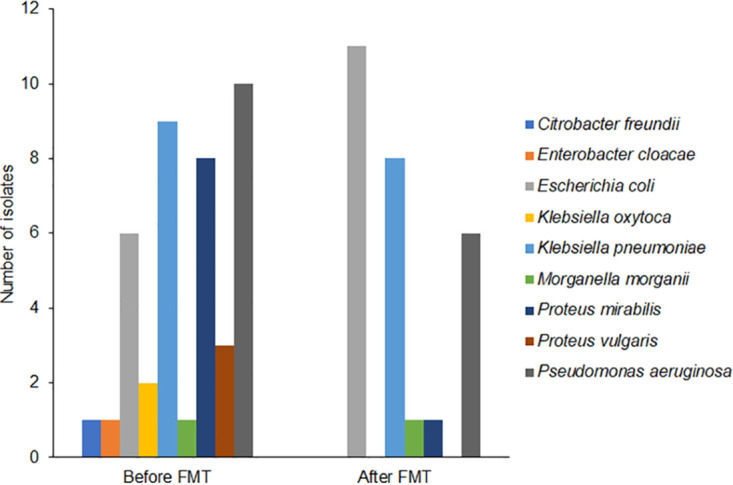
Organisms isolated from microbial cultures irrespective of culture site within 1 year before and 1 year after FMT. Total numbers of organisms before and after FMT were 41 and 28, respectively.

The majority of Gram-negative cultures for our study population were from urine samples (82%), followed by blood (6.5%), respiratory tract (i.e., bronchial alveolar lavage [BAL] fluid, mini-BAL fluid, or sputum), intraabdominal abscess (3.2%), and feces (1.6%) samples; nasopharyngeal and skin/soft tissue sites were not represented. The total numbers of organisms isolated were 41 and 28 for pre- and post-FMT samples, respectively. All organisms were from the family *Enterobacteriaceae* or the family *Pseudomonadaceae.* Escherichia coli, *Klebsiella* species, and Pseudomonas aeruginosa comprised the majority of pre- and post-FMT samples (Fig. 2). Interestingly, *Proteus* species, which were well represented in the pre-FMT samples, were not observed post-FMT, and E. coli appeared at relatively enriched levels in the post-FMT samples.

Of the remaining 262 patients, 197 did not have available Gram-negative microbiological culture data before and after FMT; 35 had Gram-negative culture data before but not after FMT within our query period; 12 had Gram-negative cultures after but not before FMT within our query period; and 6 patients with ≥2 FMTs had no Gram-negative culture data in one or more of the periods before their first FMT, after their last FMT, or between FMTs and were excluded from the study (Fig. 1). Of the 35 patients that had pre-FMT but not post-FMT Gram-negative culture data, 15 had cultures which were positive after FMT but which did not meet the inclusion criteria; those cultures had no appreciable growth or had growth of non-Gram-negative organisms or had polymicrobial growth more consistent with contamination than with infection.

### Reduction in number of positive cultures following FMT.

**FIG 3:**
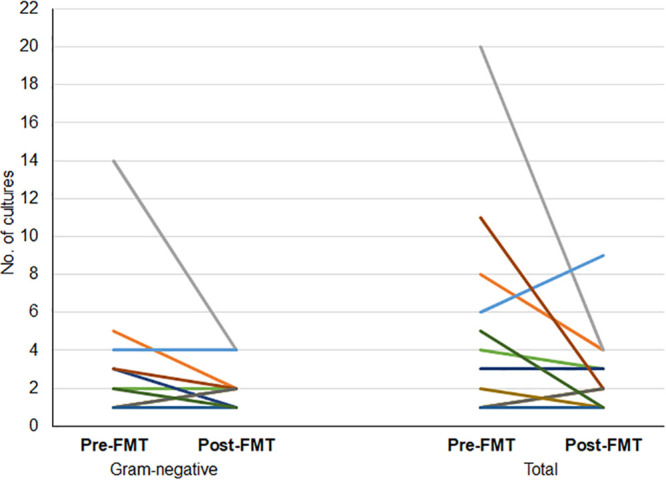
Frequency of positive cultures, Gram-negative only versus total number. Graph shows the number of cultures 1 year before and 1 year after fecal microbiota transplantation. Each line represents 1 patient.

From the 12 patients who met the inclusion criteria, there were 63 total cultures collected in the year before FMT and 34 total cultures collected in the year after FMT. With regard to positive Gram-negative cultures, there was an absolute reduction, with the number of positive cultures being 38 and 24 before and after FMT, respectively (see Table S1 in the supplemental material). In the year following FMT, the number of positive Gram-negative cultures decreased for 5 of 12 patients (41.7%), was unchanged for 4 of 12 patients (33.3%), and increased for 3 of 12 patients (25%) (Fig. 3).

10.1128/mSphere.00853-20.1 TABLE S1 Number of total and Gram-negative cultures before and after FMT for each of the 12 case series patients. Download Table S1, DOCX file, 0.01 MB. Copyright © 2020 Steed et al. 2020 Steed et al. This content is distributed under the terms of the Creative Commons Attribution 4.0 International license.

### Influence of FMT on health care provider behavior.

**FIG 4:**
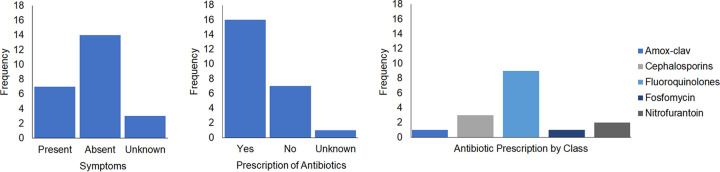
Analysis of the 24 Gram-negative cultures obtained 1 year after FMT stratified by frequency of symptom correlation and antibiotic prescription. For the instances where antibiotics were prescribed, frequency of antibiotic prescription by class was also stratified. Amox-clav, amoxicillin-clavulanic acid.

As a potential explanation for the reduced number of cultures post-FMT, we also examined whether FMT changed health care provider behavior for these patients—specifically, whether a history of FMT affected provider culture ordering or antibiotic prescribing practices. We found no evidence to suggest that a history of FMT changed culture ordering practices. Of the 24 Gram-negative cultures collected in the year following FMT, 14 were collected when the patients had no symptoms consistent with infection (Fig. 4; see also Table S2). There were several instances in which providers explicitly documented their reticence to prescribe antibiotics for asymptomatic infection to patients with a complex history of CDI and FMT administration. However, the documented concerns regarding the risk of precipitating RCDI were not exemplified in the antibiotic prescribing practices. Of the 24 post-FMT cultures, 7 were ordered on the basis of symptoms of infection, which were documented, and the patients were treated with antibiotics. Concerning the 14 cultures from asymptomatic patients, 7 of the patients were ultimately treated with antibiotics and 7 did not receive antibiotic therapy. For 3 cultures, the presence or absence of associated symptoms was not documented. For 6 of the 7 cultures from asymptomatic patients who did not receive antibiotic therapy, an alternative noninfectious diagnosis or infectious disease consultation was specifically documented (Table S2). In the instances in which antibiotics were prescribed, fluoroquinolones were heavily favored (*n* = 9/16, 56.3%) (Fig. 4).

10.1128/mSphere.00853-20.2 TABLE S2 Characteristics of the 24 Gram-negative cultures obtained 1 year after FMT, including symptom correlation, prescription of antibiotics, and narrative of changes in antibiotic prescribing practices. Download Table S2, DOCX file, 0.02 MB. Copyright © 2020 Steed et al. 2020 Steed et al. This content is distributed under the terms of the Creative Commons Attribution 4.0 International license.

### Clinical Gram-negative antimicrobial susceptibility results before and after FMT.

**FIG 5:**
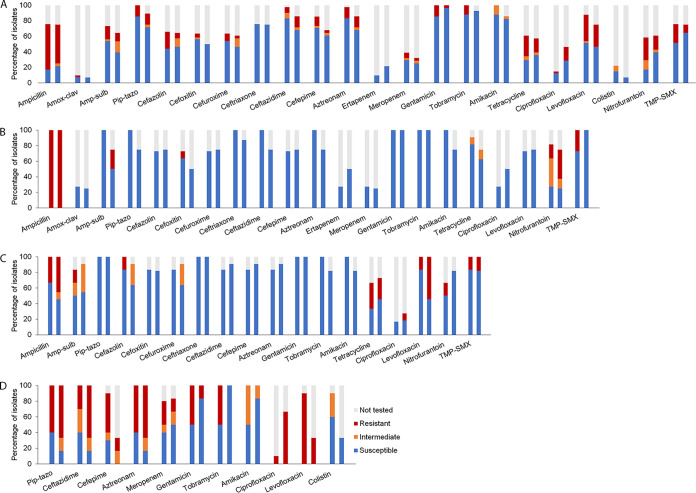
Susceptibility patterns of organisms isolated. Panel A shows the aggregate susceptibility pattern for all organisms irrespective of species. Panels B to D show the susceptibility patterns stratified by the 3 most common organisms isolated*: Klebsiella* spp. (B), E. coli (C), and P. aeruginosa (D). For panels A to D, there are paired bars for each antimicrobial drug; the first and second bars represent data before and after FMT, respectively. For clarity, for panels B to D, antimicrobial drugs that were not tested or lacked susceptibility testing for either before or after FMT were excluded. The colors represent the proportion that was not tested (gray) and the proportion that was resistant (red), the proportion that was intermediate (orange), and the proportion that was susceptible (blue) to a given antimicrobial. Abbreviations: amox-clav, amoxicillin-clavulanic acid; amp-sulb, ampicillin-sulbactam; pip-tazo, piperacillin-tazobactam; TMP-SMX, trimethoprim-sulfamethoxazole.

Across all taxa, we observed increased frequencies of susceptibility to nitrofurantoin, trimethoprim-sulfamethoxazole, and the aminoglycosides in post-FMT isolates compared to pre-FMT isolates (Fig. 5A; see also Table S3). Among the 41 Gram-negative isolates collected before FMT, 17 (41.5%) were resistant to nitrofurantoin, 10 (24.4%) were resistant to trimethoprim-sulfamethoxazole, 6 (14.6%) were resistant to gentamicin, 5 (12.2%) were resistant to tobramycin, and 5 (12.2%) were resistant to amikacin. Among the 28 Gram-negative isolates collected after FMT, only 6 (21.4%) were resistant to nitrofurantoin, 3 (10.7%) were resistant to trimethoprim-sulfamethoxazole, 1 (3.6%) was resistant to gentamicin, 0 (0%) were resistant to tobramycin, and 1 (3.6%) was resistant to amikacin.

10.1128/mSphere.00853-20.3 TABLE S3 Susceptibility profiles of pooled isolates before and after FMT. Download Table S3, DOCX file, 0.02 MB. Copyright © 2020 Steed et al. 2020 Steed et al. This content is distributed under the terms of the Creative Commons Attribution 4.0 International license.

Stratified at the species level for the most common organisms, 11 samples were positive for *Klebsiella* species pre-FMT and 8 samples were positive for *Klebsiella* species post-FMT, 6 samples were positive for E. coli pre-FMT and 11 samples were positive for E. coli post-FMT, and 10 samples were positive for P. aeruginosa species pre-FMT and 6 samples were positive for P. aeruginosa species post-FMT (Tables S4, S5, and S6). For the *Klebsiella* species, there appeared to be increased resistance to ampicillin/sulbactam and increased susceptibility to trimethoprim-sulfamethoxazole following FMT (Fig. 5B). For the E. coli isolates, we observed increased resistance to levofloxacin and increased sensitivity to nitrofurantoin following FMT (Fig. 5C) Finally, for the P. aeruginosa isolates, there appeared to be increased susceptibility to the aminoglycosides (Fig. 5D). Detailed resistance patterns are in Tables S4, S5, and S6.

10.1128/mSphere.00853-20.4 TABLE S4 Susceptibility profiles of *Klebsiella* species isolates before and after FMT. Download Table S4, DOCX file, 0.02 MB. Copyright © 2020 Steed et al. 2020 Steed et al. This content is distributed under the terms of the Creative Commons Attribution 4.0 International license.

10.1128/mSphere.00853-20.5 TABLE S5 Susceptibility profiles of E. coli isolates before and after FMT. Download Table S5, DOCX file, 0.02 MB. Copyright © 2020 Steed et al. 2020 Steed et al. This content is distributed under the terms of the Creative Commons Attribution 4.0 International license.

10.1128/mSphere.00853-20.6 TABLE S6 Susceptibility profiles of P. aeruginosa isolates before and after FMT. Download Table S6, DOCX file, 0.01 MB. Copyright © 2020 Steed et al. 2020 Steed et al. This content is distributed under the terms of the Creative Commons Attribution 4.0 International license.

## DISCUSSION

In this retrospective case series of antimicrobial susceptibilities in Gram-negative clinical cultures in the year after FMT compared to the year prior, we observed a reduction of total and Gram-negative cultures, a change in the relative proportions of Gram-negative bacteria, and an improved profile of susceptibility to nitrofurantoin, trimethoprim-sulfamethoxazole, and the aminoglycosides. FMT has been demonstrated to be highly effective at treating RCDI as a therapeutic strategy for restoring healthy intestinal microbiota, and this report adds evidence to support prospective study of FMT for MDRO decolonization and improvement of antimicrobial susceptibility.

Given that FMT has been shown to be efficacious at eradicating MDRO infection at GI and non-GI sites, we pooled all Gram-negative infections irrespective of body site cultured and found that there was an absolute decrease in the number of Gram-negative cultures post-FMT. While our case series profiles only a small population consisting of 12 patients, this finding could be consistent with a variety of different explanations, including limited follow-up, resolution of infection, reduction of Gram-negative infection or colonization following successful FMT, or changes in health care provider behavior. It is possible that a greater number of cultures were sent pre-FMT because the diagnosis of CDI was unclear, prompting a broad infectious work-up. Though all 12 patients had cultures inclusive of our 1-year pre-FMT and 1-year post-FMT time frame, two patients were essentially lost to follow-up prior to 1 year post-FMT. Subject ID 2 was followed only up until 4 months post-FMT, after which the subject subsequently enrolled in a hospice, and subject ID 169 was no longer seen at our institution after 7 months post-FMT. Interestingly, we did have a subset of 35 patients with Gram-negative culture data prior to FMT but no Gram-negative microbiological data in the 1-year period following FMT, which ultimately excluded them. Fifteen of these patients did have cultures 1 year post-FMT that did not grow a Gram-negative organism (e.g., that grew Gram-positive organisms or fungi or exhibited polymicrobial growth that was not further identified to the species level and that was more compatible with contamination), which might be consistent with reduction of Gram-negative infection or colonization following FMT. Looking at the total number of cultures ordered, a decrease in the number of cultures post-FMT (34 versus 63 pre-FMT) in the query time period might suggest clinical improvement or fewer instances of clinically significant infection that warranted an infectious work-up.

To determine whether changes in health care provider behavior contributed to the reduction of cultures post-FMT, we also evaluated whether a history of FMT resulted in changes in health care provider behavior with respect to culture ordering or antibiotic prescribing practices in the post-FMT period. We did not find any documentation to suggest that a history of FMT was considered when ordering cultures. Interestingly, 14 of 24 (58.3%) Gram-negative post-FMT cultures were drawn in the absence of symptoms and 8 of these asymptomatic cultures were urine cultures drawn as part of surveillance labs for renal transplant patients. While there was scarce information regarding the benefit of asymptomatic bacteriuria (ASB) screening and treatment in the renal transplant population during our study time period, recent guidelines now recommend against screening for or treating ASB in renal transplant recipients (30).

We observed several instances of health care providers documenting their reservations with respect to prescribing antibiotics for asymptomatic cultures due to concerns for RCDI. Despite this, post-FMT asymptomatic cultures were frequently treated with antibiotics in 7 of 14 episodes. A clear indication for therapy was documented in just one instance, for an anticipated urological procedure. There are a host of factors that contribute to antimicrobial prescribing practices (31); even with the recognition that these cultures were asymptomatic and not reflective of true infection, a positive culture result was frequently followed by prescription of antibiotics. In three of these cases which involved urinary specimens, health care providers documented that he or she would await the culture finalization before ultimately pursuing antibiotic therapy even though guidelines indicate that it is only the presence or absence of symptoms that distinguishes ASB from a urinary tract infection (UTI). In 6 of the remaining 7 asymptomatic cultures that were not treated with antimicrobial agents, either there was a compelling alternative diagnosis or the infectious disease service was consulted and recommended no antibiotics. This underscores the value of expert consultation as well as antibiotic stewardship challenges encountered in the post-FMT period.

Along with the change in the number of Gram-negative cultures post-FMT, we also identified a change in the relative proportions of Gram-negative bacteria isolated from these cultures. While E. coli, *Klebsiella* species, and P. aeruginosa comprised the majority of the pre- and post-FMT samples, the proportions of both *Klebsiella* and P. aeruginosa were decreased in the post-FMT samples whereas the proportion of E. coli was increased. As previously mentioned, *Proteus* species were abundant pre-FMT and not represented in the post-FMT period in our study. Despite the small sample size, this was unexpected given *Proteus*’s multiple adaptive features that enable its persistent colonization (32). MDR P. mirabilis in particular would be expected to have a distinct colonization advantage in this regard given its less frequent colonization clearance compared to other MDR Gram-negative bacilli (11).

Although *Proteus* spp. are typically considered pathobionts in the gastrointestinal (GI) tract, their abundance as a proportion of the microbial community is normally quite low (33). FMT recipients have been shown to experience a significant increase in gut microbiota diversity, typically shifting in composition toward the profile of the corresponding stool donor (29), and it may be that the loss of *Proteus* species post-FMT is a consequence of the restructuring of the recipient microbiome to resemble donor microbiota diversity. In a non-IBD population, there was shown to be a post-FMT decrease in the relative abundances of several bacterial genera, including *Klebsiella* and *Proteus*, which may reflect a change in gut microbial composition trending toward that of the donor microbiota (16). The etiology for the unexpected relative enrichment of E. coli post-FMT is unclear. We cannot exclude the possibility that these patients became colonized or infected with E. coli in the post-FMT period by happenstance. While there have been cases of transmission of extended-spectrum-beta-lactamase (ESBL)-producing E. coli following FMT as well as of transmission of multiple E. coli pathotypes (34), donor stool sequencing data for these samples are not available to determine whether this is reflective of the donor microbiome.

In this case series, microbiological data were pooled without respect to anatomic culture site. However, the vast majority (82%) of cultures were performed on urine samples likely related to the ease of collection of this sample and to the composition of our study population, 50% of whom carried a diagnosis of recurrent UTI. This finding adds evidence contributing to the idea of the potential value of FMT for treatment of recurrent urinary tract infections, a condition for which multiple ongoing clinical studies are evaluating its safety and efficacy (35).

When pooled, the Gram-negative isolates in this study showed increased susceptibility to nitrofurantoin and the aminoglycosides after FMT. Nitrofurantoin and the aminoglycosides share a reputation for having a minimal effect on the intestinal flora, and their use may be especially germane in a RCDI patient population. Culture-dependent and culture-independent techniques have demonstrated that nitrofurantoin minimally perturbs the composition of intestinal microbiota (36, 37), and in a study utilizing an aminoglycoside-based outpatient UTI treatment protocol for post-FMT RCDI patients, aminoglycoside administration had little impact on gut microbiota, likely related to its minimal penetration into the gut lumen (38).

In our investigation of health care provider behavior, fluoroquinolones were preferentially ordered when antibiotics were prescribed in the 1-year post-FMT period for our 12 patients. A review of the Emory University Hospital inpatient antibiogram during our study time period showed that 83% to 97% of Gram-negatives in this study would have been susceptible to levofloxacin. While many antibiotics increase risk of C. difficile-associated disease, fluoroquinolones are among those most consistently associated with such an outcome (39, 40). Our observations regarding the antimicrobial susceptibilities of the pooled post-FMT isolates may support the empirical prescription of alternative antibiotics that could be equally efficacious and could present less risk for RCDI infection. Given that antibiotic-mediated disruption of intestinal microbiota has been associated with the development of MDRO colonization and infection, the findings described above support the feasibility of using “gut-sparing” antibiotics post-FMT as a means of safeguarding the restored microbiome and halting the syndrome of recurrent cycles of infection to which prolonged antibiotic exposure and consequent gut dysbiosis may predispose patients.

Our study had important limitations due to its small number of subjects and its nonrandomized observational nature, which prevent us from drawing definitive conclusions. Given its retrospective design, we were limited in that pre- and post-FMT stool cultures were not available for microbiota analysis, which could contribute to potential detection bias. We employed an agnostic analytic approach to review microbiologic data irrespective of taxonomy or anatomic site. This approach was based on literature demonstrating that intestinal MDRO colonization typically precedes infection at other sites and on reports of eradication of extraintestinal infections after FMT, patient-level interspecies transfer of resistance elements, and reduced AR gene abundance after FMT. Future prospective studies utilizing microbiome sequencing would support further validation of this approach. To further investigate the influence of FMT on health care provider behavior, utilization of a before-and-after study design with measurement of the changes in rates of specimens sent for culture and susceptibility testing and antibiotic prescriptions or inclusion of a control group of patients with RCDI and Gram-negative infections in which CDI resolved with only antibiotics might lead to more conclusive findings. As an exploratory study, this case series suggests a possible reduction in Gram-negative infection after successful FMT. In a prospective cohort study of patients treated with FMT for RCDI matched by propensity scores to patients treated with RCDI treated with antibiotics, the FMT-treated group had a 23% lower risk of bloodstream infection after FMT and 32% increased survival (41). Prospective, controlled trials comparing patients who have undergone FMT for RCDI to patients treated only with antibiotics are needed to understand the potential benefits of FMT for reduction in non-C. difficile recurrent or invasive bacterial infection.

Here, we observed a reduction in Gram-negative cultures and an improvement in the antimicrobial susceptibility profile of Gram-negative bacteria in a small RCDI population. FMT has demonstrated broader applicability to infections outside C. difficile infections and is a viable therapeutic strategy in the fight against antibiotic resistance.

## MATERIALS AND METHODS

### Patient selection.

This was a single-center retrospective chart review study of RCDI patients who underwent FMT at Emory University between July 2012 and March 2017. Retrospective chart review was approved by the Emory University Institutional Review Board (IRB approval 71277). The FMT protocol has been approved for use at Emory Healthcare by the Medical Practices, Infection Prevention, Antibiotic Utilization, and Pharmacy and Therapeutics committees since July 2012. All patients were clinically diagnosed with refractory C. difficile infection. Informed consent was obtained prior to treatment. Demographic and clinical data were collected by chart extraction. We retrospectively identified patients with available Gram-negative culture data at any body site in the year preceding and the year following FMT. Gram-negative culture positivity was defined as any culture that grew Gram-negative bacteria.

### Data collection.

We recorded patient demographics, CDI history, count and route of FMT doses, frequency of Gram-negative culture, and associated microbiological data, including bacterial identification and susceptibility profiles of clinical isolates and site of infection, from the available Gram-negative clinical cultures in the year before and the year after FMT. In cases of multiple FMT administrations, we recorded culture data from the year before the first FMT dose and the year after the last FMT dose. We reported antimicrobial susceptibilities only for those antibiotics available on the Emory University Hospital formulary. In accordance with the Emory Medical Laboratory Policy on Antimicrobial Susceptibility Testing, piperacillin-tazobactam susceptibility was not routinely reported for Serratia marcescens; ampicillin/sulbactam susceptibility was not routinely reported for *Citrobacter*, *Enterobacter*, *Serratia*, *Pantoea*, and Cronobacter sakazakii; and meropenem susceptibility was routinely reported only for *Citrobacter*, *Enterobacter*, *Morganella*, *Serratia*, and extended-spectrum-beta-lactamase (ESBL)-positive phenotypes.

### Analytic methods.

Demographic and clinical variables were summarized using descriptive statistics. We chose to analyze the available Gram-negative microbiological data irrespective of body site or Gram-negative species isolated for the following two primary reasons. (i) The human intestinal tract is an important reservoir for MDRO colonization and AR genes (19, 20). As most mechanisms of horizontal gene transfer are not taxon restricted, we analyzed the preponderance of phenotypic AR data pre- and post-FMT irrespective of bacterial species as a surrogate for carriage of AR elements in general. (ii) Intestinal bacterial colonization with MDRO frequently precedes systemic infection at extraintestinal sites, and FMT has been shown to eradicate MDRO colonization at extraintestinal sites such as the urinary tract, respiratory tract, and wounds (35, 42–46).
