# Supplementary material for: Gram-Negative Taxa and Antimicrobial Susceptibility after Fecal Microbiota Transplantation for Recurrent Clostridioides difficile Infection
Source: mSphere. 2020 Oct 14;5(5):e00853-20. doi: 10.1128/mSphere.00853-20 (PMC7565895; doi:10.1128/mSphere.00853-20)
Supplement: TABLE S1 [file mSphere.00853-20-st001.docx]

|  | Before FMT cultures | |  | After FMT cultures | |
| --- | --- | --- | --- | --- | --- |
| Subject ID | Gram-negative only | Total |  | Gram-negative only | Total |
| 1 | 1 | 1 |  | 2 | 2 |
| 2 | 5 | 8 |  | 2 | 4 |
| 3 | 14 | 20 |  | 4 | 4 |
| 68 | 1 | 1 |  | 2 | 2 |
| 79 | 4 | 6 |  | 4 | 9 |
| 92 | 2 | 4 |  | 2 | 3 |
| 166 | 3 | 3 |  | 1 | 3 |
| 169 | 3 | 11 |  | 2 | 2 |
| 202 | 1 | 1 |  | 2 | 2 |
| 226 | 1 | 2 |  | 1 | 1 |
| 230 | 1 | 1 |  | 1 | 1 |
| 232 | 2 | 5 |  | 1 | 1 |
| **Totals** | **38** | **63** |  | **24** | **34** |
