# Supplementary material for: Gram-Negative Taxa and Antimicrobial Susceptibility after Fecal Microbiota Transplantation for Recurrent Clostridioides difficile Infection
Source: mSphere. 2020 Oct 14;5(5):e00853-20. doi: 10.1128/mSphere.00853-20 (PMC7565895; doi:10.1128/mSphere.00853-20)
Supplement: TABLE S2 [file mSphere.00853-20-st002.docx]

| Subject ID | Post-FMT gram-negative culture number | Source | Organism | Symptomatic? | Antibiotics prescribed? | Prescriber practice |
| --- | --- | --- | --- | --- | --- | --- |
| 1 | 1 | Urine | *K. pneumoniae* | Y | Y | Prescribed ciprofloxacin, which was discontinued after 3 days due to symptom resolution and desire to reduce antibiotic exposure. Symptoms recurred and patient was re-cultured 15 days later (see next row, culture 2) |
|  | 2 | Urine | *K. pneumoniae* | Y | Y | Prescribed fosfomycin |
| 2 | 1  2 | Respiratory  Respiratory | *K. pneumoniae*  *K. pneumoniae, P. aeruginosa* | N  N | N  N | Patient presented with malaise, chronic cough at baseline, low grade temperature. Bronchoscopy performed to investigate pneumonia versus Churg-Strauss exacerbation. Cultures 1 and 2 collected during same admission. Infectious Disease consulted. Felt to be colonization due to long-standing severe bronchiectasis. Risk of systemic antibiotics and rCDI recognized to be high. No antibiotics prescribed in order to reduce antibiotic exposure |
| 3 | 1 | Urine | *K. pneumoniae* | N | Y | Patient asymptomatic, UA and culture drawn as part of renal transplant surveillance labs. Abnormal UA noted but antibiotics not prescribed until culture finalization due to absence of symptoms. Prescribed cephalexin. |
|  | 2 | Urine | *K. pneumoniae* | N | Y | Outside UA and culture drawn as part of renal transplant surveillance labs. Prescribed ciprofloxacin by transplant nephrologist and referred to Infectious Disease for recurrent UTIs in setting of complex CDI history. Infectious disease physician was concerned about repeatedly treating urinary tract colonization and precipitating MDR infection or rCDI, recommended treatment only if symptomatic and to consider antibiotic rotation if so |
|  | 3 | Urine | *E. coli* | N | N | UA and culture ordered by geriatric psychiatrist. Results forwarded to Infectious Disease for assistance with antibiotic management. In conjunction with Transplant Nephrologist, antibiotics not prescribed due to low WBC/hpf and absence of symptoms |
|  | 4 | Urine | *E. coli* | N | N | UA and culture drawn as part of renal transplant surveillance labs. Patient asymptomatic and positive culture attributed to colonization. No antibiotic prescribed to reduce antibiotic exposure; low serum WBC and low UA WBC/hpf cited to support this decision |
| 68 | 1 | Urine | *K. pneumoniae* | N | Y | Patient had presented to the Emergency Department with rectal bleeding, no endorsed infectious symptoms. Patient was called 3 days later with the results of positive urine culture and was prescribed nitrofurantoin |
|  | 2 | Urine | *E. coli* | N | N | Patient presented with upper abdominal pain, no urinary symptoms. UA and culture drawn as part of triage labs. Patient eventually diagnosed and managed for small bowel obstruction.  Recognition of positive urine culture not noted though patient with known enterovesical fistula. No antibiotics prescribed. |
| 79 | 1 | Urine | *K. pneumoniae, P. mirabilis* | N | Y | Outside UA and culture drawn as part of renal transplant surveillance labs. Patient’s partner called and expressed concern about antibiotic as she recalled being advised to avoid antibiotics post-fecal transplant. Transplant nephrologist felt that it was far enough out from his fecal transplant (6 months) that antibiotic therapy should not be harmful. Patient was prescribed ciprofloxacin |
|  | 2 | Urine | *K. pneumoniae* | Presumed not as patient underwent elective urological procedure | Y | Outside UA and culture drawn as part of renal transplant surveillance labs. Transplant nephrologist prescribed amoxicillin-clavulanic acid 3 days later. Patient underwent cystoscopy with Urology on same day as antibiotic prescription sent. Unclear if antibiotics ordered in anticipation of urological procedure. |
|  | 3 | Urine | *P. aeruginosa, P. aeruginosa* | Unknown | Unknown | Outside UA and culture drawn as part of renal transplant surveillance labs. Unknown if patient symptomatic or prescribed antibiotics |
|  | 4 | Urine | *P. aeruginosa, P. aeruginosa* | Unknown | Y | Outside UA and culture drawn as part of renal transplant surveillance labs. Patient prescribed ciprofloxacin |
| 92 | 1 | Urine | *E. coli* | Unknown | Y | Reason for specimen collection or presence of symptoms not documented. Prescribed ciprofloxacin as UA felt to be consistent with infection (positive leukocyte esterase, nitrites, 366 WBC/hpf) |
|  | 2 | Urine | *E. coli* | Y | Y | Patient called to request UA as she was concerned she might be having a “bladder infection”. Prescribed ciprofloxacin |
| 166 | 1 | Urine | *M. morganii* | N | Presumed none | UA and culture drawn as part of renal transplant surveillance labs. Labs with abnormalities, including acidosis and acute kidney injury; patient sent to ED and discharged following IV hydration. Abnormal UA noted. Transplant nephrologist preferred to await urine culture finalization before antibiotic prescription given history of recalcitrant CDI with caveat that antibiotics would be prescribed if culture grew >50,000 CFU/mL. Culture resulted with 10,000-40,000 CFU/mL so antibiotics presumed not given. |
| 169 | 1  2 | Urine  Urine | *E. coli*  *E. coli* | Y  Y | Y  Y | Patient presented with decreased PO intake, failure to thrive, and mild dysuria. There was concern that culture 1 was not drawn cleanly (>100 WBC/hpf) and so culture 2 was drawn as a straight-cath specimen (only 12 WBC/hpf). Patient resisted antibiotic initiation citing history of rCDI and so antibiotics held until finalization of urine culture. Patient given one dose ceftriaxone, discharged with cefpodoxime. Infectious Disease curb-sided who agreed with management plan. |
| 202 | 1 | Urine | *E. coli* | N | Y | Patient admitted for alcohol withdrawal. Recognition of positive urine culture not noted but she received levofloxacin, later switched to moxifloxacin for suspected pneumonia. |
|  | 2 | Urine | *S. marescens* | Y | Y | Patient admitted again for alcohol withdrawal but exhibited hematuria and endorsed dysuria. Prescribed ciprofloxacin |
| 226 | 1 | Tissue | *E. coli* | N | N | Patient reported pain/ulcerations at stoma site. Ulcerative lesion had not responded to antibiotic therapy in the past and so lesion was thought to be less likely infectious in origin and diagnosis favored to be cutaneous Crohn’s disease versus pyoderma gangrenosum. No antibiotics prescribed. |
| 230 | 1 | Urine | *E. coli* | N | Y | Patient was prescribed nitrofurantoin. Patient expressed concerns regarding *C. difficile* recurrence but prescribing physician documented that nitrofurantoin was selected as it mostly concentrates in the urine and has less of a systemic effect |
| 232 | 1 | Urine | *E. coli* | Y | Y | Patient endorsed urinary frequency. Prescribed ciprofloxacin |
| Abbreviations: UA: urinalysis, UTI: urinary tract infection | | | | | | |
