# Supplementary material for: Gram-Negative Taxa and Antimicrobial Susceptibility after Fecal Microbiota Transplantation for Recurrent Clostridioides difficile Infection
Source: mSphere. 2020 Oct 14;5(5):e00853-20. doi: 10.1128/mSphere.00853-20 (PMC7565895; doi:10.1128/mSphere.00853-20)
Supplement: TABLE S3 [file mSphere.00853-20-st003.docx]

| Antimicrobial drug | All organisms (No. of isolates, %) | | | | |
| --- | --- | --- | --- | --- | --- |
|  | Before FMT (n = 41) | |  | After FMT (n = 28) | |
|  | S | R |  | S | R |
| Ampicillin | 7 (17.1) | 24 (58.5) |  | 6 (21.4) | 15 (53.6) |
| Amoxicillin-clavulanic acid | 3 (7.3) | 1 (2.4) |  | 2 (7.1) | 0 (0) |
| Ampicillin-sulbactam | 22 (53.7) | 9 (19.5) |  | 11 (39.3) | 7 (25.0) |
| Piperacillin-tazobactam | 35 (85.4) | 6 (14.6) |  | 20 (71.4) | 5 (17.9) |
| Cefazolin | 18 (43.9) | 9 (22.0) |  | 13 (46.4) | 5 (17.8) |
| Cefoxitin | 23 (56.1) | 3 (7.3) |  | 14 (50) | 0 (0) |
| Cefuroxime | 22 (53.7) | 4 (9.8) |  | 13 (46.4) | 4 (14.3) |
| Ceftriaxone | 31 (75.6) | 0 (0) |  | 21 (75) | 0 (0) |
| Ceftazidime | 34 (82.9) | 6 (14.6) |  | 19 (67.9) | 5 (17.9) |
| Cefepime | 29 (70.7) | 6 (14.6) |  | 17 (60.7) | 2 (7.1) |
| Aztreonam | 34 (82.9) | 6 (14.6) |  | 19 (67.9) | 5 (17.9) |
| Ertapenem | 4 (9.8) | 0 (0) |  | 6 (21.4) | 0 (0) |
| Meropenem | 12 (29.3) | 4 (9.8) |  | 7 (25) | 2 (7.1) |
| Gentamicin | 35 (85.4) | 6 (14.6) |  | 27 (96.4) | 1 (3.6) |
| Tobramycin | 36 (87.8) | 5 (12.2) |  | 26 (92.9) | 0 (0) |
| Amikacin | 36 (87.8) | 5 (12.2) |  | 23 (82.1) | 1 (3.6) |
| Tetracycline | 12 (29.3) | 13 (31.7) |  | 10 (35.7) | 6 (21.4) |
| Ciprofloxacin | 5 (12.2) | 1 (2.4) |  | 8 (28.6) | 5 (17.9) |
| Levofloxacin | 21 (51.2) | 15 (34.6) |  | 13 (46.4) | 8 (28.6) |
| Colistin | 6 (14.6) | 3 (7.3) |  | 2 (7.1) | 0 (0) |
| Nitrofurantoin | 7 (17.1) | 17 (41.5) |  | 11 (39.3) | 6 (21.4) |
| Trimethoprim-sulfamethoxazole | 21 (51.2) | 10 (24.4) |  | 18 (64.3) | 3 (10.7) |
| Abbreviation: FMT, fecal microbiota transplantation; S, susceptible; R, resistant  Intermediate susceptibilities were considered resistant and were included under this category | | | | | |
