# Supplementary material for: Gram-Negative Taxa and Antimicrobial Susceptibility after Fecal Microbiota Transplantation for Recurrent Clostridioides difficile Infection
Source: mSphere. 2020 Oct 14;5(5):e00853-20. doi: 10.1128/mSphere.00853-20 (PMC7565895; doi:10.1128/mSphere.00853-20)
Supplement: TABLE S4 [file mSphere.00853-20-st004.docx]

| Antimicrobial drug | *Klebsiella spp* (No. of isolates, %) | | | | |
| --- | --- | --- | --- | --- | --- |
|  | Before FMT (n = 11) | |  | After FMT (n = 8) | |
|  | S | R |  | S | R |
| Ampicillin | 0 (0) | 11 (100) |  | 0 (0) | 8 (100) |
| Amoxicillin/clavulanic acid | 3 (27.3) | 0 (0) |  | 2 (25) | 0 (0) |
| Ampicillin/Sulbactam | 11 (100) | 0 (0) |  | 4 (50) | 2 (25) |
| Piperacillin/Tazobactam | 11 (100) | 0 (0) |  | 6 (75) | 0 (0) |
| Cefazolin | 8 (72.7) | 0 (0) |  | 6 (75) | 0 (0) |
| Cefoxitin | 7 (63.6) | 1 (9.1) |  | 4 (50) | 0 (0) |
| Cefuroxime | 8 (72.7) | 0 (0) |  | 6 (75) | 0 (0) |
| Ceftriaxone | 11 (100) | 0 (0) |  | 7 (87.5) | 0 (0) |
| Ceftazidime | 11 (100) | 0 (0) |  | 6 (75) | (0) |
| Cefepime | 8 (72.7) | 0 (0) |  | 6 (75) | 0 (0) |
| Aztreonam | 11 (100) | 0 (0) |  | 6 (75) | 0 (0) |
| Ertapenem | 3 (27.3) | 0 (0) |  | 4 (50) | 0 (0) |
| Meropenem | 3 (27.3) | 0 (0) |  | 2 (25) | 0 (0) |
| Gentamicin | 11 (100) | 0 (0) |  | 8 (100) | 0 (0) |
| Tobramycin | 11 (100) | 0 (0) |  | 8 (100) | 0 (0) |
| Amikacin | 11 (100) | 0 (0) |  | 6 (75) | (0) |
| Tetracycline | 9 (81.8) | 1 (9.1) |  | 5 (62.5) | 1 (12.5) |
| Ciprofloxacin | 3 (27.3) | 0 (0) |  | 4 (50) | 0 (0) |
| Levofloxacin | 8 (72.7) | 0 (0) |  | 6 (75) | 0 (0) |
| Nitrofurantoin | 3 (27.3) | 6 (54.5) |  | 2 (25) | 4 (50) |
| Trimethoprim/sulfamethoxazole | 8 (72.7) | 3 (27.3) |  | 8 (100) | 0 (0) |
| Abbreviation: FMT, fecal microbiota transplantation; S, susceptible; R, resistant  Intermediate susceptibilities were considered resistant and were included under this category | | | | | |
