# Supplementary material for: Gram-Negative Taxa and Antimicrobial Susceptibility after Fecal Microbiota Transplantation for Recurrent Clostridioides difficile Infection
Source: mSphere. 2020 Oct 14;5(5):e00853-20. doi: 10.1128/mSphere.00853-20 (PMC7565895; doi:10.1128/mSphere.00853-20)
Supplement: TABLE S5 [file mSphere.00853-20-st005.docx]

| Antimicrobial drug | *E. coli* (No. of isolates, %) | | | | |
| --- | --- | --- | --- | --- | --- |
|  | Before FMT (n = 6) | |  | After FMT (n = 11) | |
|  | S | R |  | S | R |
| Ampicillin | 4 (66.7) | 2 (33.3) |  | 5 (45.5) | 6 (54.5) |
| Ampicillin-sulbactam | 3 (50) | 2 (33.3) |  | 6 (54.5) | 4 (36.3) |
| Piperacillin-tazobactam | 6 (100) | 0 (0) |  | 11 (100) | (0) |
| Cefazolin | 5 (83.3) | 1 (16.7) |  | 7 (63.6) | 3 (27.3) |
| Cefoxitin | 5 (83.3) | 0 (0) |  | 9 (81.8) | 0 (0) |
| Cefuroxime | 5 (83.3) | 0 (0) |  | 7 (63.6) | 3 (27.3) |
| Ceftriaxone | 6 (100) | 0 (0) |  | 11 (100) | (0) |
| Ceftazidime | 5 (83.3) | 0 (0) |  | 10 (90.9) | 0 (0) |
| Cefepime | 5 (83.3) | 0 (0) |  | 10 (90.9) | 0 (0) |
| Aztreonam | 5 (83.3) | 0 (0) |  | 10 (90.9) | 0 (0) |
| Ertapenem | ---*^a^* | --- |  | 1 (9.1) | 0 (0) |
| Gentamicin | 6 (100) | 0 (0) |  | 11 (100) | 0 (0) |
| Tobramycin | 6 (100) | 0 (0) |  | 9 (81.8) | 0 (0) |
| Amikacin | 6 (100) | 0 (0) |  | 9 (81.8) | 0 (0) |
| Tetracycline | 2 (33.3) | 2 (33.3) |  | 5 (45.5) | 3 (27.3) |
| Ciprofloxacin | 1 (16.7) | 0 (0) |  | 2 (18.2) | 1 (9.1) |
| Levofloxacin | 5 (83.3) | 1 (16.7) |  | 5 (45.5) | 6 (54.5) |
| Nitrofurantoin | 3 (50) | 1 (16.7) |  | 9 (81.8) | 0 (0) |
| Trimethoprim-sulfamethoxazole | 5 (83.3) | 1 (16.7) |  | 9 (81.8) | 2 (18.2) |
| *^a^*Not tested  Abbreviation: FMT, fecal microbiota transplantation; S, susceptible; R, resistant  Intermediate susceptibilities were considered resistant and were included under this category | | | | | |
