# Supplementary material for: Gram-Negative Taxa and Antimicrobial Susceptibility after Fecal Microbiota Transplantation for Recurrent Clostridioides difficile Infection
Source: mSphere. 2020 Oct 14;5(5):e00853-20. doi: 10.1128/mSphere.00853-20 (PMC7565895; doi:10.1128/mSphere.00853-20)
Supplement: TABLE S6 [file mSphere.00853-20-st006.docx]

| Antimicrobial drug | *P. aeruginosa* (No. of isolates, %) | | | | |
| --- | --- | --- | --- | --- | --- |
|  | Before FMT (n = 10) | |  | After FMT (n = 6) | |
|  | S | R |  | S | R |
| Piperacillin-tazobactam | 4 (40) | 6 (60) |  | 1 (16.7) | 5 (83.3) |
| Ceftazidime | 4 (40) | 6 (60) |  | 1 (33.3) | 5 (66.7) |
| Cefepime | 3 (30) | 6 (60) |  | 0 (0) | 2 (33.3) |
| Aztreonam | 4 (40) | 6 (60) |  | 1 (16.7) | 5 (83.3) |
| Meropenem | 4 (40) | 4 (40) |  | 3 (50) | 2 (33.3) |
| Gentamicin | 5 (50) | 5 (50) |  | 5 (83.3) | 1 (16.7) |
| Tobramycin | 5 (50) | 5 (50) |  | 6 (100) | 0 (0) |
| Amikacin | 5 (50) | 5 (50) |  | 5 (83.3) | 1 (16.7) |
| Ciprofloxacin | 0 (0) | 1 (10) |  | 0 (0) | 4 (66.7) |
| Levofloxacin | 0 (0) | 9 (90) |  | 0 (0) | 2 (33.3) |
| Colistin | 6 (60) | 3 (30) |  | 2 (33.3) | 0 (0) |
| Abbreviation: FMT, fecal microbiota transplantation; S, susceptible; R, resistant  Intermediate susceptibilities were considered resistant and were included under this category | | | | | |
